# Optical Absorption Enhancement in CdTe Thin Films by Microstructuration of the Silicon Substrate

**DOI:** 10.3390/ma10060607

**Published:** 2017-06-01

**Authors:** Jesús Rangel-Cárdenas, Hugo Sobral

**Affiliations:** Centro de Ciencias Aplicadas y Desarrollo Tecnológico, Universidad Nacional Autónoma de México (CCADET-UNAM), Apartado Postal 70-186, Ciudad de México 04510, Mexico; jesus.arc@gmail.com

**Keywords:** thin films, optical properties, laser materials processing, microstructure fabrication

## Abstract

In this work, the reflectance, optical absorption, and band gap have been determined for CdTe thin films grown on planar and microstructured substrates. The treated surface was prepared by laser ablation of a silicon wafer, forming holes in a periodic arrangement. Thin films were grown by pulsed laser ablation on silicon samples kept at 200 °C inside a vacuum chamber. The presence of CdTe was verified with X-ray diffraction and Raman spectroscopy indicating a nanocrystalline zinc blended structure. The optical absorption of thin films was calculated by using the Fresnel laws and the experimental reflectance spectrum. Results show that reflectance of 245 nm films deposited on modified substrates is reduced by up to a factor of two than the obtained on unchanged silicon and the optical absorption is 16% higher at ~456 nm. Additionally, it was determined that the band gap energy for planar and microstructured films is about 1.44 eV for both cases.

## 1. Introduction

Cadmium telluride is a compound with potential applications in various science fields, such as photovoltaic devices [1], light emitting diodes [2,3], solar cells [4,5], X-ray and gamma detectors [6]**,** or even biological applications [7,8]. This wide range of applications is mainly due to its optical properties and the energy band gap value, allowing an efficient conversion from sunlight to electric energy. CdTe thin films have a high optical absorption coefficient of about 10^4^ cm^–1^, absorbing about 92% of the visible light in a thickness of just 1 µm. This efficiency is well above crystalline silicon, which needs about 200 µm to reach the same absorption value [9]. Therefore, it is possible to employ CdTe thin films to produce efficient solar cells with reduced recombination losses and improved short-circuit current density. A further benefit of thin films is that they can be synthesized by several physical and chemical methods, such as chemical bath deposition [10], spray pyrolysis [11], thermal evaporation [12], electrodeposition [13,14], sputtering [15], and pulsed laser ablation (PLD) [16,17,18]. The latter allows the in situ fabrication of solar cells with a precise control of the film width of about 0.2 nm, and can be scaled up for commercialization. Furthermore, PLD allows the preservation of stoichiometry when adequate conditions of temperature and pressure are satisfied. 

On the other hand, several methods were employed to improve the light absorptance of CdTe thin films, including antireflection coatings [19], employing nanostructured materials [20], and surface plasmon resonance [21,22], among others. All these enhancement techniques are based on light trapping. Furthermore, different geometries have been used to improve light absorption, when the incident angle is different to the normal. Periodic structures in the micro- and nano-scale were suggested as light-trapping textures to confine light inside the material. Surface modification is commonly made on silicon substrates by plasma etching [23,24]. The structures used to enhance light absorption include nanowires [25], cones [26,27], pyramids [28], cosine surface arrays [29], and Fourier-series-based periodic arrays [30]. Another scheme to increase light absorption by thin films is reported in [31]. Here, a honeycomb microstructure was prepared using a wet-chemical method that, in turn, increases the number of beam reflections on the modified material. Most of these surface modifications were employed to enhance solar cell efficiency. For instance, in the work of Nalwa and Chaudhary [27] an average increment in photonic absorption of 14% for the visible range was calculated, using a 150 nm thick active layer on a microstructured material.

In this work, we propose an alternative method to increase the light absorption efficiency by substrate microstructuration with pulsed laser ablation. This surface processing avoids sample contamination in comparison to other chemical methods. Modification was carried out by concentrating the laser emission with a micro-lens array onto a silicon surface to produce a symmetric microstructured arrangement. Afterward, CdTe thin films were grown over the preheated substrate by pulsed laser deposition. Thin film quality has been investigated by X-ray diffraction and Raman spectroscopy. Furthermore, the absorption coefficient enhancement and the band gap were obtained for thin films deposited on planar and microstructured substrates.

## 2. Materials and Methods

### 2.1. Substrate Modification

**Figure 1:**
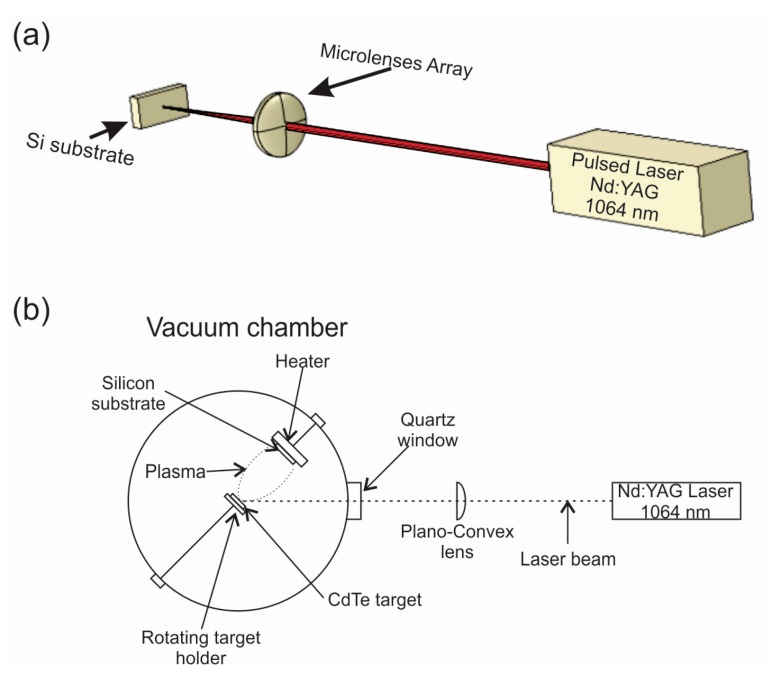
(**a**) Experimental scheme employed for microstructure silicon substrates; (**b**) Schematic arrangement used for CdTe thin film synthesis by the pulsed laser deposition technique.

Thin films were deposited on two types of silicon substrates. One of them was a 1 × 1 cm^2^ planar commercial silicon wafer and the other was previously microstructured by using pulsed laser ablation to produce a regular pattern (see Figure 1a). The employed laser was an Nd:YAG (Continuum, Surelite III, San Jose, CA, USA), emitting 5 ns pulses at 1064 nm with a frequency of 10 Hz and an energy of 200 mJ per pulse. The emission was focused by a 10 × 10 mm^2^ micro-lens arrays with focal lengths of 3.5 and 12 cm. Microstructures were formed with different numbers of pulses, but the best results, taking into account the absorption coefficient, were obtained for 2000 pulses.

### 2.2. Thin Film Synthesis

Cadmium telluride thin films were grown by using pulsed laser deposition. The experimental scheme diagram is shown in Figure 1b. Ablation of a high purity 99.99% CdTe target (from Lesker) was performed with 50 mJ pulses at a frequency of 10 Hz. The target was placed inside a vacuum chamber kept at ca. 10^−5^ Torr and pressure was monitored by using Pirani and cold cathode gauges (Oerlikon Leybold Vacuum, D-50968 Köln and Leybold 157 51-D PR 35, Cologne, Germany). The laser beam was focused onto the target surface by a 30 cm plano-convex lens at an incidence angle of 45° with a fluence of 8 J/cm^2^. This value was obtained by measuring the crater diameter at the surface level with a profilometer (Sloan/Veeco, Dektak IIA, Planview, NY, USA). The CdTe target was rotated at 10 rpm to avoid crater formation and to maintain the ablation conditions throughout the disposition process. The deposition time was 30 minutes, corresponding to 18,000 pulses. The substrate was kept at 200 °C during the film deposition and was located at 10 cm opposite to the target.

### 2.3. Characterization of CdTe Thin Films

Thin film thickness was measured by a profilometer and observed by an optical microscope. During the deposition, part of the substrate was covered, leaving a step on the synthesized film, making it possible to measure its thickness. CdTe presence and crystalline structure were determined by X-ray diffraction (Rigaku Ultima-IV, Bragg-Brentano geometry, Tokyo, Japan) with Cu Kα radiation (λ = 0.154 nm). A diffractogram scan was collected with a grazing incidence angle of 1.5° over a 2θ range of 20°–70°.

Furthermore, a micro-Raman spectrometer with a 3 µm spatial resolution (Thermo Fisher Scientific DXR2, Waltham, MA, USA), was used to investigate the deposited thin films focusing the microscope on different spatial regions. Two excitation wavelengths, 532 and 780 nm, were used and the obtained peaks were adjusted by a Lorentzian function. 

Optical properties of thin films were further investigated by measuring the specular and diffuse reflectance. This was accomplished through an integrating sphere housed in a spectrophotometer (Cary 5000, Agilent Technologies, Santa Clara, CA, USA) in the 400–800 nm wavelength range. From the obtained results, the optical absorption coefficient and the energy gaps for films grown on both types of substrates were calculated.

## 3. Results and Discussion

### 3.1. Optical Microscopy

**Figure 2:**
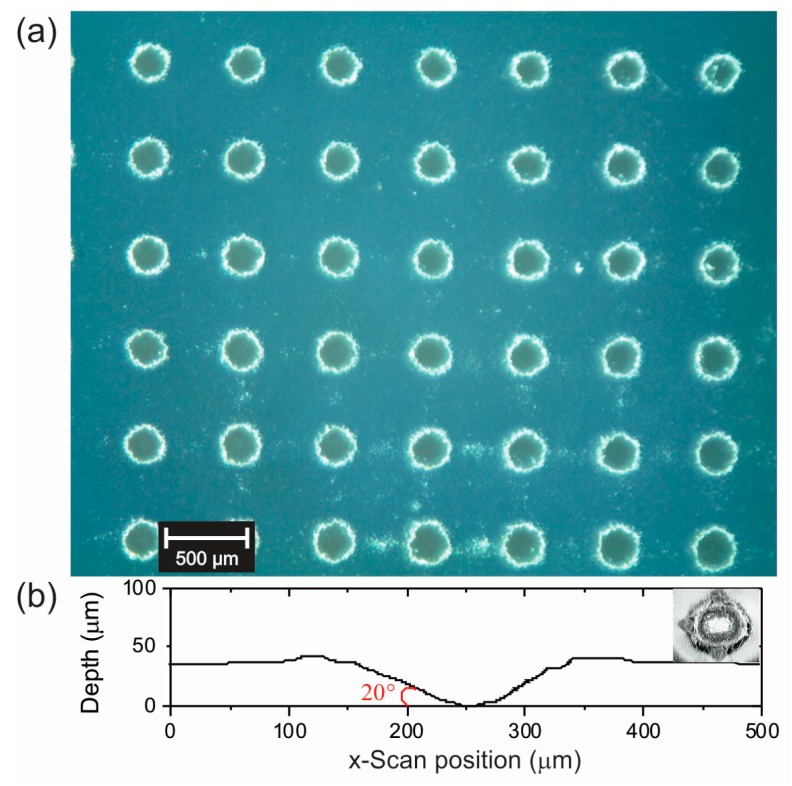
(**a**) Optical microscopy of a CdTe thin film deposited over a microstructured substrate; and (**b**) profilometry measurement corresponding to a typical hole: 35 µm depth and 181 µm width.

Figure 2a shows an image of the substrate microstructuration produced by laser ablation with the 12 cm focal length micro-lens array. Under these conditions, a regular pattern of holes separated about 580 µm between centers was obtained. Profilometer measurements show that each crater is about 180 µm wide at the surface level and 35 µm deep (see Figure 2b). Additionally, it was determined that the CdTe film thickness is about 245 nm for both planar and microstructured films.

### 3.2. X-ray Diffraction Analysis

**Figure 3:**
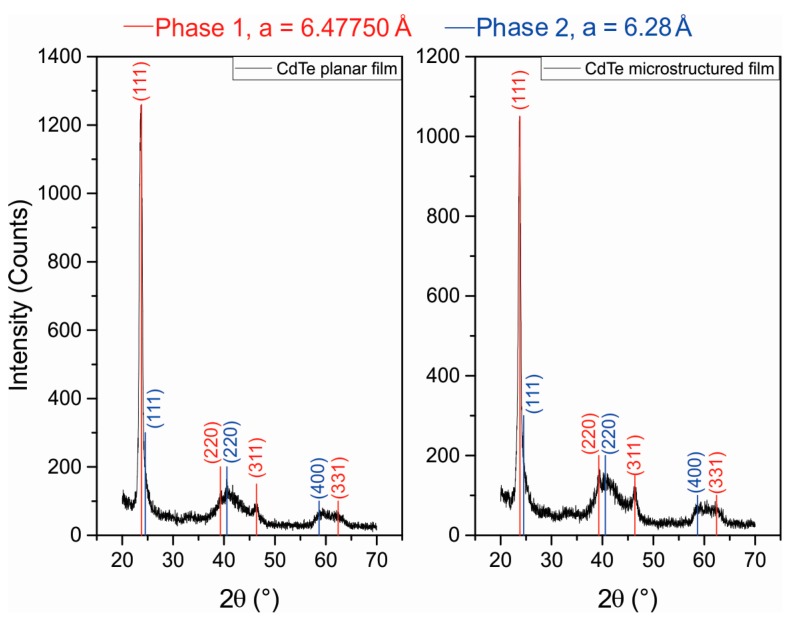
X-ray diffraction pattern for planar (**a**) and microstructured (**b**) CdTe films. Both films present two cubic phases with lattice parameters of 6.48 and 6.28 Å.

An X-ray diffractogram of CdTe thin films is shown in Figure 3. Thin films exhibit two cubic phases with lattice parameters (*a*) of 6.48 and 6.28 Å for both planar and microstructured film cases (ICDD data base PDF 03-065-0890 and 03-065-1046). The main phase with a lattice constant of 6.48 Å is in good agreement with that reported for the CdTe bulk [32]. However, a minority second phase was also observed with a lattice parameter that differed by 0.20 Å with respect to the main one. This may be due to the kinetic energy of the ablated particles due to the laser Gaussian spatial profile of the laser beam and the recrystallization process during the deposition process. 

The interplanar distance *d_hkl_* was calculated from the peak positions and the Bragg’s law:

(1) $$n\lambda =2d_{hkl}\sin\theta$$

where *λ* is the incident X-ray wavelength; *θ* is the angle of incidence, and n is the order of diffraction. The cubic system follows the relation:

(2) $$a=d_{hkl}\sqrt{h^{2}+k^{2}+l^{2}}$$

which allows us to obtain the Miller indices (*hkl*) of the lattice planes. For the phase 1 (*a* = 6.4775 Å), the respective prominent peaks correspond to reflections from the (111), (220), (311), and (331) planes and, for phase 2 (*a* = 6.28 Å), from (111), (220), and (400).

From the diffraction selection rules, when *h + k, h + l*, and *k + l* are even numbers, a zinc blended structure was obtained. Diffraction peaks for other compounds, such as metallic Cd, Te, or their oxides states, were not observed.

The crystallite size (D) in the CdTe films was estimated from Scherrer equation:

(3) $$D=\frac{0.9\lambda }{\beta \cos\theta }$$

where *β* is the line broadening at full width at half maximum intensity (FWHM). The calculated crystallite sizes are 11.4 ± 4 nm, thereby indicating the nanocrystalline nature of the film.

Hence, the deposited CdTe thin films are polycrystalline in nature due to the presence of sharp structural peaks, with a zinc blended structure and preferential orientation in the (111) plane.

### 3.3. Raman Spectroscopy

**Figure 4:**
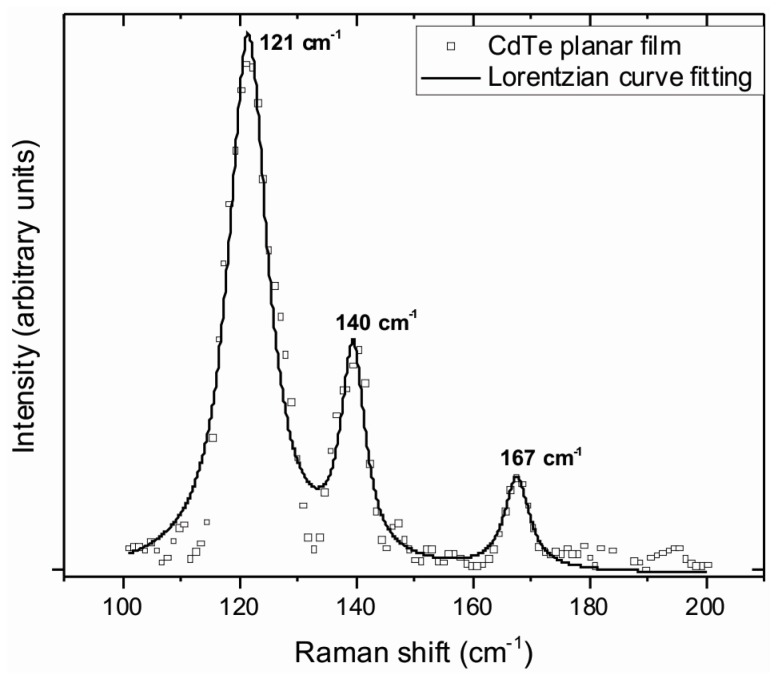
Raman spectrum for a planar CdTe film.

CdTe Raman spectra were recorded for the planar substrate using a 532 nm excitation wavelength in the frequency range of 100–200 cm^−1^. Measured peaks were fitted with a Lorentzian profile, to investigate the quality of CdTe synthesized films. Results shown in Figure 4 display three emission peaks. The first at 121 cm^–1^ corresponding to the A1 mode of the tellurium Raman active peak [33]. The second one at 140 cm^–1^ was assigned to a combination of the tellurium peak E and the transversal optic (TO) phonon of the CdTe located at 139 and 141 cm^–1^, respectively [34]. The third observed peak, located at 167 cm^–1^, is associated with the CdTe longitudinal optical (LO) phonon.

**Figure 5:**
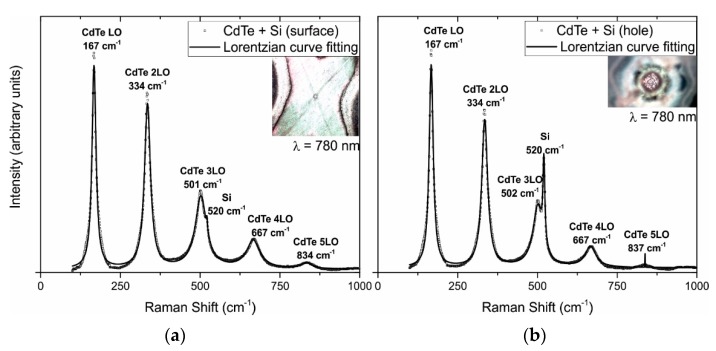
Raman spectrum for a microstructured thin film deposited over silicon, focused over the surface (**a**) and at the bottom of a hole (**b**). Inserts: images of the regions where the signals were acquired.

Spectra measured on the microstructured silicon substrates were obtained by using a 780 nm excitation wavelength in the range of 100 to 1000 cm^−1^ (see Figure 5). For these samples, the laser was focused in two different spatial regions: on top of the film surface (a) and inside the ablated holes (b), to verify the presence of CdTe over the whole microstructured substrate. For both regions, Raman spectra show a peak at 167 cm^−1^, which corresponds to the CdTe LO phonon [35]. Additionally, it can be observed that the first harmonic at 335 cm^−1^ and, subsequently, the second, third, and fourth harmonics at 501, 667, and 834 cm^−1^, respectively. These results are in agreement with those reported in [36,37,38]. Furthermore, the characteristic peak of silicon at 520 cm^−1^ [39] was also detected. 

### 3.4. Optical Properties

**Figure 6:**
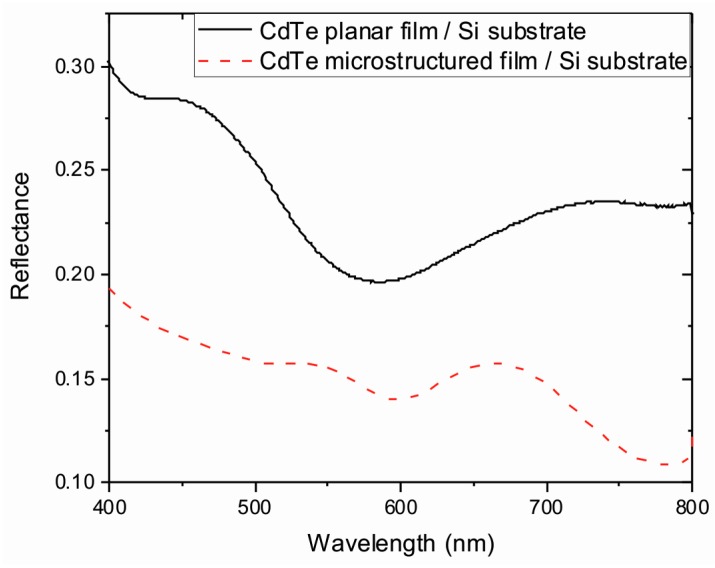
Reflectance as a function of wavelength obtained for planar and microstructured CdTe films deposited on a silicon substrate.

The energy absorbed by the films was determined from reflectance measurements as a function of the wavelength R(λ) (see Figure 6). As expected, the reflectance presents a series of maxima and minima values due to light interference caused by the reflection on both interfaces of the film. For the planar film, a minimum around 585 nm was observed, which is in agreement with the theoretical expected values using the Fresnel equations for a 245 nm thick CdTe film on a silicon substrate [40]. Furthermore, the minimum reflectance value expected was about 0.24, which is slightly higher than the measured value. This small deviation with the predicted values can be attributed to a non-homogenous layer. On the other hand, results show that the reflectance of planar films is up to 1.5-fold larger than the values obtained for microstructured thin films. The reduction in the measured reflectance for microstructured substrates can be attributed to light reabsorption of reflected light inside the burned holes [31].

The absorption coefficient *α*, was determined from the experimental results by using Beer’s law:

(4) $$\alpha (\lambda )=\frac{A(\lambda )}{d}$$

where *d* is the film thickness. Here, the sample optical absorbance *A*(*λ*) is given by

**Figure 7:**
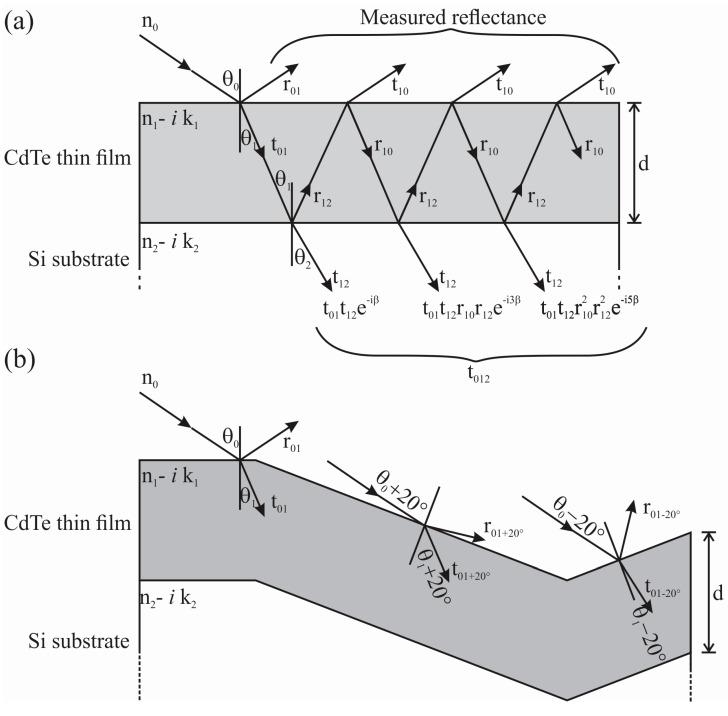
Optical interference for a thin film formed on a substrate (ambient/thin film/substrate) (**a**) and for a film formed on a microstructured substrate (**b**). *n*_0_ is the refractive index of air; *n*_1_ − *i k*_1_ and *n*_2_ − *i k*_2_ are the complex refractive index of the film and substrate, respectively; *r_jk_* and *t_jk_* are the amplitude of the reflection and transmission coefficients.

(5) A(λ)=1−R(λ)−T(λ)

where *T*(*λ*) is the transmittance through the film. However, *T*(*λ*) could not be experimentally measured since the substrate is opaque. Thus, the transmission can be calculated by using the Fresnel equations for a thin film formed on a planar substrate [41]. In our case, the light travels from air with a refractive index *n*_0_ and through the CdTe film with thickness d and transmitted to the silicon substrate; the film and the substrate have complex refractive indexes *n*_1_ − *i k*_1_ and *n*_2_ − *i k*_2_, respectively. Figure 7a shows a diagram of the light path with the substrate have infinite thickness being assumed. From the Fresnel equations for the transverse electric s and magnetic p components it is possible to obtain [41]

(6) $$\begin{array}{cc} r_{jk,p}=\frac{(n_{k}-ik_{k})\cos\theta_{j}-(n_{j}-ik_{j})\cos\theta_{k}}{(n_{k}-ik_{k})\cos\theta_{j}+(n_{j}-ik_{j})\cos\theta_{k}}, & r_{jk,s}=\frac{(n_{j}-ik_{j})\cos\theta_{j}-(n_{k}-ik_{k})\cos\theta_{k}}{(n_{j}-ik_{j})\cos\theta_{j}+(n_{k}-ik_{k})\cos\theta_{k}} \end{array}$$

(7) $$\begin{array}{cc} t_{jk,p}=\frac{2(n_{j}-ik_{j})\cos\theta_{j}}{(n_{k}-ik_{k})\cos\theta_{j}+(n_{j}-ik_{j})\cos\theta_{k}}, & t_{jk,s}=\frac{2(n_{j}-ik_{j})\cos\theta_{j}}{(n_{j}-ik_{j})\cos\theta_{j}+(n_{k}-ik_{k})\cos\theta_{k}} \end{array}.$$

where *r_jk_* and *t_jk_* are the amplitude of reflection and transmission coefficients at each interface, correspondingly. The values for the complex refractive index n and k for the substrate and the film have been obtained from the experimental data reported in [42,43].

The amplitude transmission coefficient is obtained from the sum of all of the transmitted waves:

(8) $$t_{012}=t_{01}t_{12}e^{-i\beta }+t_{01}t_{12}r_{10}r_{12}e^{-i3\beta }+t_{01}t_{12}r_{10}^{2}r_{12}^{2}e^{-i5\beta }+\cdots .$$

The phase variation between the surface and the interface *β* can be written as

(9) $$\beta =\frac{2\pi d}{\lambda }(n_{1}-ik_{1})\cos\theta_{1}=\frac{2\pi d}{\lambda }{\left[{\left(n_{1}-ik_{1}\right)}^{2}-{\left(n_{0}-ik_{0}\right)}^{2}\sin^{2}\theta_{0}\right]}^{1/2}.$$

Since the infinite series $y=a+ax+ax^{2}+ax^{3}+\cdots$, is reduced to y=a/(1−x) and from Equation (6), *r*_10_ = −*r*_01_; then, substituting into Equation (8), it is possible to write

(10) $$t_{012}=\frac{t_{01}t_{12}e^{-i\beta }}{1+r_{01}r_{12}e^{-i2\beta }}.$$

Thus, the amplitude transmission coefficients for p- and s-polarized waves are expressed by

(11) $$\begin{array}{cc} t_{012,p}=\frac{t_{01,p}t_{12,p}e^{-i\beta }}{1+r_{01,p}r_{12,p}e^{-i2\beta }} & t_{012,s}=\frac{t_{01,s}t_{12,s}e^{-i\beta }}{1+r_{01,s}r_{12,s}e^{-i2\beta }} \end{array}$$

and the transmittance for p-and s-polarized waves is given by

(12) $$\begin{array}{cc} T_{p}=\frac{n_{2}\cos\theta_{2}}{n_{0}\cos\theta_{0}}{\left|t_{012,p}\right|}^{2} & T_{s}=\frac{n_{2}\cos\theta_{2}}{n_{0}\cos\theta_{0}}{\left|t_{012,s}\right|}^{2} \end{array}$$

The incidence and transmission angles at each interface can be obtained by applying Snell’s law, and the total transmittance *T* through

(13) $$T=\frac{T_{p}+T_{s}}{2}.$$

Since, for the microstructured case, all of the involved dimensions are much larger than the incident wavelengths, it is possible to use the same ray analysis. The top of the microstructured surface is planar and *T* could be obtained as above; the difference to *T* is due to the laser produced craters. We consider an increment of the area due to the holes as $\frac{A_{walls}}{A_{planar}+A_{walls}}$. For simplicity, we have assumed that the crater has a conical shape (see Figure 2b), as depicted in Figure 7b. Accordingly, the above analysis can be used by adding a term to take into account these new areas, considering the change in the angle of incidence as shown in the figure.

Hence, the amplitude transmission coefficient for the p component could be written as:

(14) $$t_{jk,p}=\frac{A_{planar}}{A_{planar}+A_{walls}}\frac{2(n_{j}-ik_{j})\cos\theta_{j}}{(n_{k}-ik_{k})\cos\theta_{j}+(n_{j}-ik_{j})\cos\theta_{k}}+\frac{A_{walls}}{A_{planar}+A_{walls}}\frac{2(n_{j}-ik_{j})\cos\theta_{j}}{(n_{k}-ik_{k})\cos\theta_{j}+(n_{j}-ik_{j})\cos\theta_{k}}.$$

**Figure 8:**
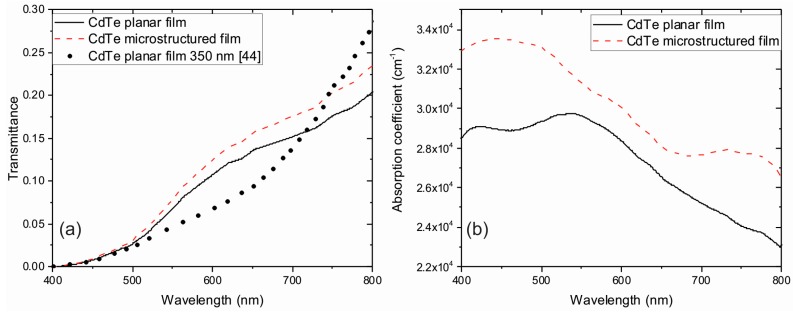
(**a**) Calculated transmittance for planar and microstructured CdTe films; thin film thickness: 245 nm. Filled circles correspond to 350 nm CdTe thin films obtained at normal incidence [44]; (**b**) Absorption coefficients for planar and microstructured CdTe films calculated using the employed model based on Fresnel equations and the transmittance experimental data.

Thus, from Equation (13) *T* has been obtained and replaced in Equations (4) and (5) to calculate the absorption coefficient *α* as a function of the wavelength (see Figure 8). For both, planar and microstructured substrates, the transmittance was calculated averaging the obtained values when the incidence angle was varied from the normal incidence by up to 70°.

It was observed that the transmittance decreases to values near zero as the extinction coefficient for CdTe grows [43]. Calculated data for films grown on planar substrates are in agreement with experimental data published elsewhere, taking into account the employed film thickness [44]. Hence, these results verify the accuracy of the refractive indices used in the simulations. Since the reflectance of films on microstructured substrates is lower than those obtained using the planar configuration (see Figure 6), then higher values for the transmittance are predictable, as those shown in Figure 8a. Furthermore, larger values for the absorption coefficient was obtained for films deposited over microstructured substrates, in the whole visible range with values, exceeding by 10% those obtained in planar films (Figure 8b). This enhancement could be attributed to a reduced reflectance due to a larger absorption area of the modified substrate and that the light travels a longer path throughout the film.

**Figure 9:**
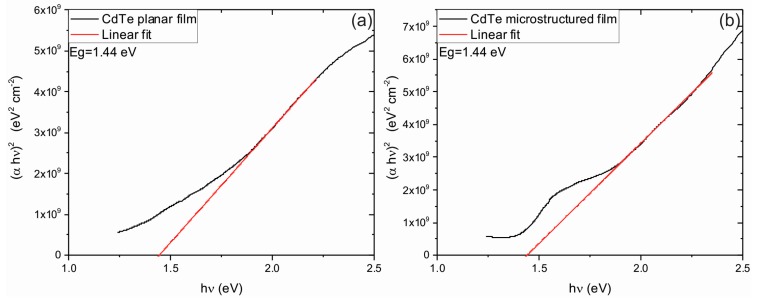
Tauc plots corresponding to planar (**a**) and microstructured (**b**) thin films. Linear fits were used to determine the optical band gaps (*E_g_*).

From the absorption coefficient data, the energy band gap *E_g_* can be obtained through

(15) $$\alpha h\nu =A{\left(h\nu -E_{g}\right)}^{n}$$

where *h* is the Plank constant, ν is the photon frequency, and A is a constant. The exponent n depends on the material transition type and could be 0.5 for a direct transition and 2 for an indirect one. In this work *n* = 0.5 since CdTe presents a direct transition as reported in [1]. Figure 9 shows the Tauc plots obtained from the experimental data. The energy band gap could be calculated by extrapolation of the linear fit at the absorption limit *α* = 0 [45].

The obtained values for the band gap were 1.44 ± 0.02 eV for films deposited on planar and microstructured substrates. Here, it is expected that the same optical band gap for films deposited on treated and untreated substrates will be obtained, since X-ray diffraction results exhibited the same crystalline structure. Furthermore, the obtained value is in agreement with previously-reported data [9,46,47]. 

**Figure 10:**
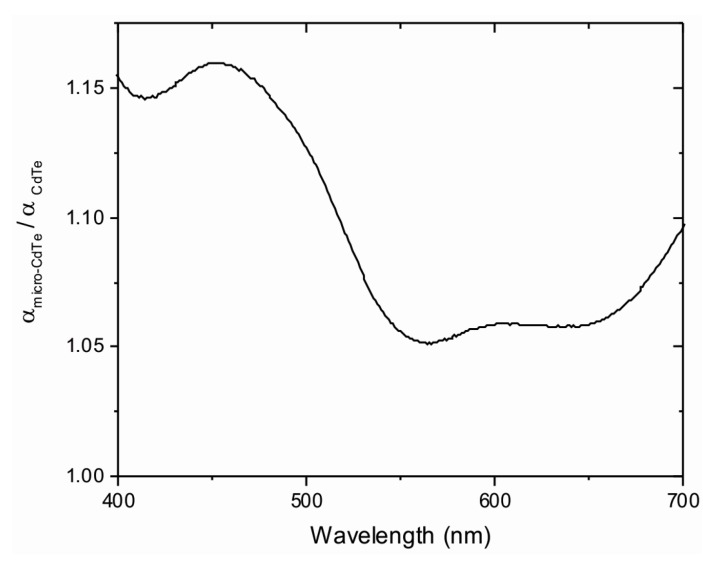
Absorption coefficient ratio between microstructured and planar films as a function of the wavelength. Data were obtained from Figure 8b.

A better comparison between both kinds of films can be obtained by dividing the optical absorption coefficient obtained for microstructured substrates by the planar ones. Figure 10 shows that microstructured films absorb 10% more on average, in comparison with films grown on planar substrates, with a maximum value of 16% at ca. 456 nm. 

The absorption coefficient depends on the absorbance, which strongly depends on the used surface modification. Therefore, the obtained value could be further enhanced by increasing the absorption area. This can be accomplished by increasing the density of holes and their depths and reducing the hole diameters. However, according to the employed model, the array periodicity does not change the obtained absorptance. A similar result was reported elsewhere [48,49], where theoretical models were used to compare the quantum efficiency for periodic and randomly-textured thin films.

## 4. Conclusions

Pulsed laser deposition has been employed to grow CdTe thin films on planar and microstructured silicon substrates. The regular microstructured arrangement of holes have been obtained using laser ablation.

Films deposited on microstructured substrates presents an absorption coefficient enhancement in the visible range of 10% on average compared to planar thin films. This can be due to a larger optical path of the modified substrates and its larger area compared to the planar ones. This value could be further enhanced by increasing the depth of the ablated craters and reducing the spacing between holes.

Since the improvement method is based only on substrate geometric modification, other thin film synthesis methods and substrates could be employed to enhance light absorption. Additionally, other hole sizes and distributions can be used (at the micrometric scale) to enhance the optical absorption due to a surface increment. Further development of this approach could be employed for photovoltaic device applications.
